# Hydrodynamic cavitation in Stokes flow of anisotropic fluids

**DOI:** 10.1038/ncomms15550

**Published:** 2017-05-30

**Authors:** Tillmann Stieger, Hakam Agha, Martin Schoen, Marco G. Mazza, Anupam Sengupta

**Affiliations:** 1 Stranski-Laboratorium für Physikalische und Theoretische Chemie, Technische Universität Berlin, Straße des 17. Juni 115, 10623 Berlin, Germany; 2 Max Planck Institute for Dynamics and Self-Organization (MPIDS), Am Faßberg 17, 37077 Göttingen, Germany; 3 Physics and Material Science Unit, University of Luxembourg, 162 Avenue de la Faiencerie, L-1511 Luxembourg, Luxembourg; 4 Department of Chemical and Biomolecular Engineering, North Carolina State University, Engineering Building I, Box 7905, 911 Partners Way, Raleigh, North Carolina 27695, USA; 5 Ralph M. Parsons Laboratory for Environmental Science and Engineering, Department of Civil and Environmental Science and Engineering, Massachusetts Institute of Technology, 15 Vassar Street, Cambridge, Massachusetts 02139, USA; 6 Institute for Environmental Engineering, Department of Civil, Environmental and Geomatic Engineering, ETH Zurich, Stefano-Franscini-Platz 5, 8093 Zurich, Switzerland

## Abstract

Cavitation, the nucleation of vapour in liquids, is ubiquitous in fluid dynamics, and is often implicated in a myriad of industrial and biomedical applications. Although extensively studied in isotropic liquids, corresponding investigations in anisotropic liquids are largely lacking. Here, by combining liquid crystal microfluidic experiments, nonequilibrium molecular dynamics simulations and theoretical arguments, we report flow-induced cavitation in an anisotropic fluid. The cavitation domain nucleates due to sudden pressure drop upon flow past a cylindrical obstacle within a microchannel. For an anisotropic fluid, the inception and growth of the cavitation domain ensued in the Stokes regime, while no cavitation was observed in isotropic liquids flowing under similar hydrodynamic parameters. Using simulations we identify a critical value of the Reynolds number for cavitation inception that scales inversely with the order parameter of the fluid. Strikingly, the critical Reynolds number for anisotropic fluids can be 50% lower than that of isotropic fluids.

## Abstract (web summary)

Cavitation is the formation of vapour bubbles within a liquid and is undesirable in many industrial applications. Here Stieger *et al*. show how the anisotropic fluids influence this process in a nematic liquid crystal and find that orientational ordering of molecules can tune the onset of cavitation.

Cavitation, the nucleation of vapour bubbles within a liquid, is one of the oldest and most extensively researched phenomena in fluid dynamics^1^,^3^,^4^. Commonly triggered by local heating of a fluid above its boiling temperature (superheating), or by physical processes that abruptly decrease the fluid's pressure below its saturated vapour pressure^5^, cavitation is often accompanied by pernicious ramifications. The drop in fluid pressure during cavitation can have considerable implications on a range of industrial and biomedical processes. Industries conventionally engaged in tribology^6^,^7^ and hydraulics of mechanical components^8^,^9^, sonochemical^10^,^11^ and cryogenic processing^12^ and high-throughput extrusion processes in polymer material plants^13^ routinely experience material degradation due to cavitation-induced wear and tear, resulting in considerable economic losses. The scope and the extent to which cavitation underpins crucial steps in biophysical and biomedical processes have recently started to be recognized. Whether it is ascent of the sap in tall trees^14^, the lubrication efficacy of thin films of synovial fluids between our joints^15^, the performance of a mechanical heart valve^16^ or the ability of shrimps to deftly employ it as a tool to stun their preys^17^, cavitation is the common functional determinant in a range of biological processes.

Cavitation sets in when liquids are subject to tensile stresses. Above a critical value, known as the breaking tension or cavitation threshold^18^, liquids experience negative pressures, a thermodynamically metastable state^19^,^20^. While in some cases reaching the cavitation threshold may be the desired goal (for example, in applications based on sonomechanics), in others, the physical and material parameters are tuned to avoid the inception of cavitation. Hydrodynamic cavitation—the cavitation in liquids induced by a hydrodynamic flow—has been the subject of both experiments and extensive simulations, including molecular dynamics (MD) simulations of Lennard–Jones fluids^21^,^22^, Monte Carlo simulations^23^,^24^,^25^ and density functional theory^26^,^27^,^28^,^29^. From inception to implosion, the life time of a cavitating bubble can depend on a number of factors: volume of dissolved gases in the liquid matrix, presence of inclusions (for example, particulate matters) or pre-existing nucleation sites (for example, gas bubbles) and roughness of the solid surfaces in contact with the liquid. Such multiparameter dependence can pose technical challenges to study cavitation experimentally—a possible explanation for the lack of corresponding investigations in anisotropic liquids.

Anisotropic liquids constitute a special class of complex, non-Newtonian liquids, in which the molecules exhibit long-range order in their orientations or positions. Common examples of such liquids include liquid crystals (LCs)^30^,^31^, polymeric liquids far from equilibrium (LC polymers)^32^ and electro/magnetorheological fluids. In such partially ordered media, the hydrodynamic properties, and outcomes, are determined not just by the inertial and viscous parameters, but also by the long-range elastic interactions between molecules, and those between the molecules and confining boundaries. Predictors of cavitation in polymeric and ordered liquids have been subject of a few simulation-based studies, including MD simulations of the nucleation of cavities in a homogeneous polymer under tensile strain^33^, and within polymers containing nanocomposites^34^. Interestingly, a number of building blocks in living organisms are anisotropic^35^,^36^,^37^,^38^: amphiphilic lipids in cellular membranes, cytoskeletal and muscle proteins and the collagens and proteoglycans of connective tissues possess intrinsic order; and biopolymer solutions in the cytoskeleton^39^ and F-actin filaments^40^,^41^ have nematic symmetry. Consequently, the study of cavitation in complex anisotropic media becomes essential, especially in light of biomedical procedures like electrohydraulic lithotripsy^42^, and shock-wave lithotripsy that can trigger intraluminal bubble expansion leading to rupture of capillaries and small blood vessels^43^.

Despite its far-reaching implications, a systematic study of cavitation in anisotropic liquids is yet to be undertaken. LCs and LC polymer materials, with their highly tunable physical properties^44^, offer a realistic substitute for biological samples to carry out investigations of cavitation within anisotropic media. To the best of our knowledge, cavitation in LC materials has been studied only in the contexts of phase transitions in LCs under negative pressure^45^, and the disruption of director alignment^46^. While in the former case, cavitation was induced by an isochoric cooling of small LC droplets embedded in a glass-forming material, acoustic waves were employed in the latter to nucleate cavitating domains. Any attempt to study hydrodynamic cavitation in LCs is still largely lacking.

In this work, we use microfluidic experiments to study hydrodynamic cavitation in a liquid crystalline material (5CB, nematic phase at room temperature, see Methods), and employ MD simulations to quantitatively capture the dynamics of cavitation inception and growth. Cavitation occurred due to the flow of nematic 5CB past a cylindrical pillar, placed within a linear microfluidic channel. A vapour–liquid interface, pinned on the micropillar surface, encases the cavitation domain. Interestingly, the inception and growth of the domain takes place in the Stokes flow regime (Reynolds number, 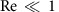), that is, under laminar flow conditions. We have identified a range of Ericksen numbers, 200<Er<500 (the Ericksen number characterizes the nematofluidic conditions of our system^44^, see Methods), in which cavitation is reproducible and stably sustained. From its first appearance at a given Ericksen number, the cavitation domain grows in size, and within a few hours, attains the saturation volume. For Ericksen numbers below the lower bound, no cavitation was observed, whereas at Er>500, the domain was unstable, and was observed to shrink in size. In all our experiments, the cavitation volume was localized around the stagnation point downstream of the micropillar. Using MD simulations, we numerically reproduce the microfluidic experiments and, by identifying the material and flow parameters, establish the physical principles that govern cavitation in anisotropic liquids. We find that the critical Reynolds number, Re_cr_ (minimum Re for cavitation inception), scales inversely with the order parameter of the anisotropic liquid. This, in contrast to corresponding flows in isotropic phase (order parameter≃0), was calculated to be less than half of that in isotropic liquids. Finally, we develop theoretical arguments based on the free energy of the fluid that support all our findings.

## Results

### Experiments

**Figure 1:**
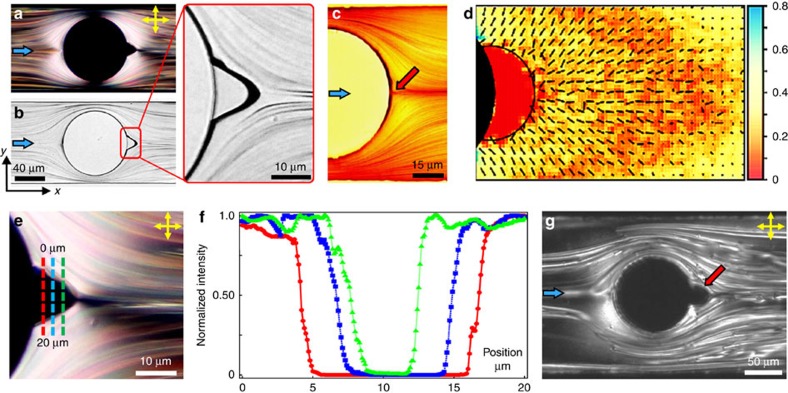
Cavitation in nematic LC due to flow past a micron-sized pillar. (**a**) Polarized optical micrograph and (**b**) white light micrograph show steady-state cavitation domain in nematic LC, 5CB. The channel and pillar surfaces have homeotropic boundary condition. Right panel: Magnified projection of the cavitating domain. (**c**) Minimum intensity projection of the scattered light over time due to flowing disclination lines. The disclinations were used to trace the streamlines of the LC flow past the micropillar. The cavitating domain is locally stabilized at the hydrodynamic stagnation point (indicated by the red arrowhead) downstream of the micron-sized pillar. For the micrographs shown here, the pillar diameter is 2*r*=80 μm, placed within a 100 μm wide and 15 μm deep microchannel. (**d**) Map of the local nematic order parameter in proximity of the cavitation domain obtained from MD simulations (see attached colour bar). The area of solid red colour corresponding to vanishing nematic order represents the cavitation. Small dashes in the maps represent the local director field. The length of a dash represents its three-dimensional orientation: shorter dashes have an orientation closer to the normal to the plane of the map. (**e**) Magnified view of the cavitating domain observed between crossed polarizers. The intensity of the transmitted light (normalized by the maximum intensity) is measured along the dashed lines: red, blue and green, and plotted in (**f**). For each line, the maximum value of the normalized intensity (bright regions) is recorded outside the cavitation domain (bulk director is oriented at 45° relative to the polarizers). At the apex of the cavitation domain and further downstream, the director is oriented parallel to the flow direction that appears dark between the crossed polarizers. The gas-filled cavitation domain also appears dark (minimum intensity) due to the total extinction of transmitted light. (**g**) Cavitation domain was also observed under planar surface anchoring conditions, visualized here between crossed polarizers.

The first appearance of the microscopic cavitation domain was detected at Er≃200. As shown in Fig. 1a,b, the vapour phase localizes at the hydrodynamic stagnation point downstream of the micron-sized pillar. As the streamlines of the flowing 5CB divide upon approaching the micropillar, both Er and Re first increase locally, reach maximum values at the point of minimum separation and reattain their original values as the flows converge downstream of the pillar. With an average flow speed of 800 μm s^−1^ (Er=200), the velocity reaches a maximum of 4,000 μm s^−1^ at the constriction (minimum distance of 10 μm between the pillar surface and the channel wall). This corresponds to Er=945 and Re=0.1, a hydrodynamic Stokes regime. The resulting drop in the hydrodynamic pressure corresponds to ≃8 mPa, corroborating that the absolute pressure decreases below the vapour pressure of nematic 5CB (≃0.1 mPa at 298 K^47^).

Figure 1c shows the downstream hydrodynamic stagnation point (red arrowhead), visualized using a differential intensity projection of the light scattered by the flowing disclinations (Methods). Due to the high Er flows, singular topological defects readily fills up the nematic bulk that scatter white light due to the lensing effect^48^. These freely flowing disclinations (both ±1/2 and ±1 defects are present) follow the streamlines, and accurately map the laminar flow field around the micropillar. Between the crossed polarizers, the cavitation domain has a dark appearance due to complete extinction of the transmitted white light. As shown in Fig. 1b, the interface between the vapour and liquid phases of 5CB is distinctly visible under white light. Cavitation was also observed within microfluidic devices possessing planar surface anchoring (Fig. 1g). Irrespective of the surface anchoring, no cavitation was observed in nematic flows at Er<200. In all cases, the nematic director field, average orientation of the LC molecules, visualized using polarization microscopy, was observed to be aligned by the flow field (nematic 5CB is a flow aligning LC^32^). This agrees very well with flow-induced director field described previously^49^.

Figure 1d maps the local nematic order in proximity of the cavitation domain, obtained through MD simulations. In agreement with the polarized optical measurements (Fig. 1e), our simulations predict that the LC molecules anchor homeotropically at the fluid–vapour interface. As shown in Fig. 1f, the normalized intensity of the transmitted light, plotted for three adjacent sections on the cavitation domain (red, blue and green dashed lines), attains maximum value just outside the cavitation domain. The maximum intensity corresponds to bulk director orientation of 45° relative to the crossed polarizers. Close to the apex of the cavitation domain, the director is oriented along the flow direction. This appears dark between crossed polarizers as the director is parallel to one of the polarizers. A minimum intensity is also observed inside the cavitation domain due to total extinction of the white light passing through the gas phase between crossed polarizers. The intermediate values of the intensity at the cavitation interface suggest that the molecules have a weak homeotropic alignment at the interface, as also confirmed by our MD simulations. Measurements on 5CB show that the molecules align homeotropically at the fluid–vapour interface^50^,^51^,^52^, in agreement with existing studies of the LC–air interface for 5CB^53^. Our MD simulations correctly capture this behaviour that lends confidence to the predictions about the underlying physics. The disclination lines found in the experiments are not present in the MD system (Fig. 1d) as they require much larger system sizes to develop.

**Figure 2:**
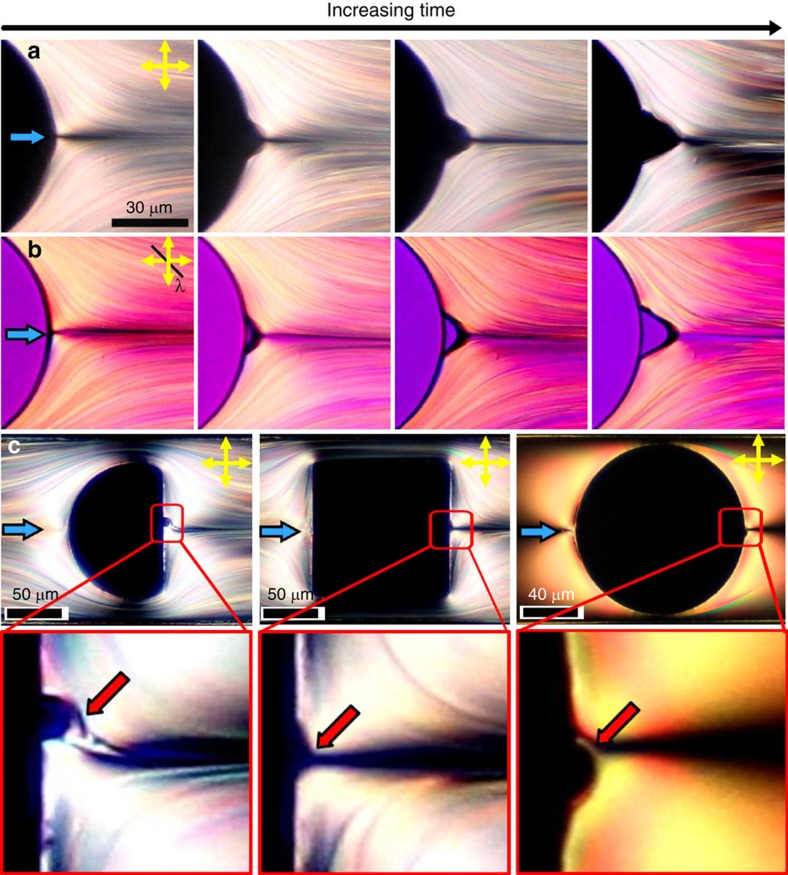
Cavitation domain growth. (**a**) Polarized optical micrographs represent the growth of the cavitation volume over time. The total time elapsed is 5 h. The minimum intensity regions (dark appearance) are observed either due to the extinction of the transmitted light as it passes through the vapour phase (in the cavitation domain), or through the bulk nematic phase aligned parallel to one of the polarizers. The two cases are distinguished using a *λ*-plate. (**b**) Introduction of the *λ*-plate confirms the absence of the nematic phase in the cavitation domain, and distinguishes it from the bulk nematic aligned parallel to the polarizer. (**c**) Cavitating domains were observed upon LC flow past different obstacle geometries: semicircle (left) and square (middle); and at different channel depths (right, *d*≈10 μm). Shallow channels require high Er numbers for cavitation to be triggered.

Upon appearance, the cavitation volume increases over time, and typically within few hours (4–7 h) of steady flow of the nematic 5CB, saturates into a constant size. Figure 2a,b show polarization optical microscopy (POM) micrographs of a cavitating domain developing over 5 h at 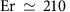. The time taken to attain the steady-state volume varies in experiments and, in addition to the roughness of the pillar surface (higher roughness promotes cavitation), it showed a qualitative dependence on the depth of the microchannel. At a given Er, it took longer to reach the saturated volume in a shallow channel (*d*<12 μm), compared with that in deeper channels. However, once formed, the cavitation domain in a shallow channel was observed to be stable for longer times, possibly due to strong pinning of the vapour–liquid interface at the top and bottom surfaces of the microchannel. The POM micrographs with *λ*-plate (Fig. 2b) confirm the absence of the nematic phase within the cavitation domain. While cavitation can be initiated by pre-existing gas bubbles entrapped in the asperities of the confining solid surfaces, we do not expect this to play a determining role in our experiments. Under similar surface conditions, no cavitation was observed when 5CB in the isotropic phase was flowed, and in experiments with deionized water. The inception of cavitation only in the nematic 5CB suggests that cavitation is triggered due to the complex anisotropic nature of the flowing material. Cavitation was also reproduced with obstacles having noncircular cross-sections, including semicircular and square geometries (Fig. 2c). At high flow speeds (Er>500), the domain volume was observed to reduce in size. This is counterintuitive, since high flow speed (high Re) is known to favour the onset of cavitation in isotropic fluids. However, in the present setting, we speculate that the strong viscous forces disrupt the pinned vapour–liquid interface, and shear off minuscule gas bubbles from the saturated cavitation domain. This can reduce the domain volume, triggering a dynamic tradeoff between the growth and decay processes that regulate the final cavitation volume at a given Er.

### Simulations

**Figure 3:**
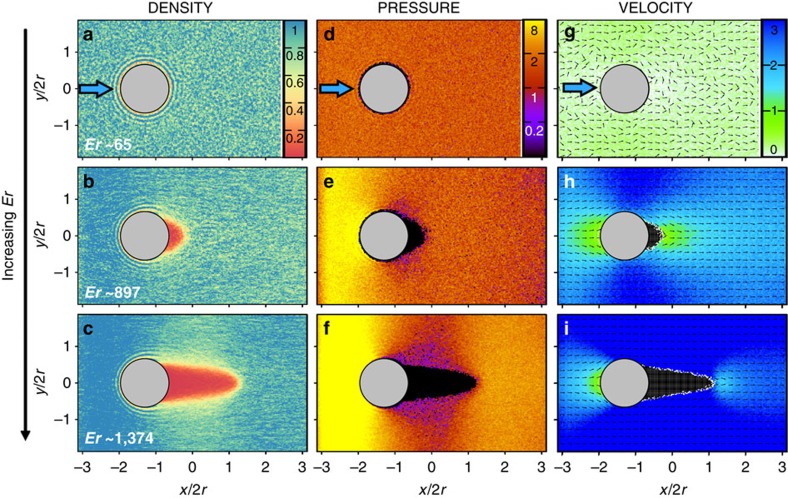
MD simulation of the hydrodynamic states. (**a**–**c**) Maps of the magnitude of local density in the *x–y* plane located at *z*=0 (see attached color bar). The circle represents the cylindrical pillar and the arrow above each column indicates the direction of flow, at different Ericksen numbers: (**a**) Er≃65, (**b**) Er≃897 and (**c**) Er≃1,374. (**d**–**f**) MD simulated local pressure plots and (**g**–**i**) corresponding local velocity fields, carried out at the same Er as in (**a**–**c**). Colour bars attached to plots in the top panel refer to all plots in the same column.

To obtain an in-depth understanding of the physical processes that underlie the cavitation phenomenon, we turn to a theoretical modelling of our experimental setup. Due to the limitations in computer performance, the length scales accessible to MD simulations are still too small compared with the micron-sized cavitation domain in our experiments. However, the MD simulations do provide us access to the early stages of the LC cavitation, in addition to the structural information that is not easily tractable with phenomenological theories of LCs (see Methods and Supplementary Information). To characterize the onset and evolution of cavitation within the LC fluid, in Fig. 3a–c we present the maps of the local density in a *x–y* cross-section of the system. For the system at rest, that is, without flow, the density is homogeneous throughout the system. For moderate flow conditions the density remains homogeneous across the system (see Fig. 3a). However, at high flow rates, starting at Er≃897, the density across the system starts to change. Figure 3c shows a well-developed cavitation domain at Er≃1,374. Specifically, we observe a drop in the density in the downstream wake of the pillar (Fig. 3b). The density of this area corresponds to that of vapour phase, and therefore demonstrates hydrodynamic cavitation. The size of the vapour domain first increases till Er≃1,374, and then grows at a reduced rate. At Er≃1,586 the cavitation domain reaches the maximum size and does not grow further.

We notice that the Er at which cavitation initiates is about a factor of four higher than in our experiments. This apparent discrepancy is resolved by estimating the nematodynamic parameters locally around the micron-sized obstacle, rather than the far-field values. Locally, the Er reaches much higher values due to the increased flow speed through the constriction between the pillar and channel walls^44^. When cavitation first occurs in our experiments, at a far-field Er of 200 (*v*=800 μm s^−1^), the corresponding local Ericksen number Er_loc_=945 (*v*_loc_=4,000 μm s^−1^). This value is intriguingly close to Er=897 obtained in the MD simulations. We note that the local Reynolds number still falls within the Stokes regime 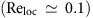. The close agreement between the experiments and simulations prompts us to conclude that the microfluidic experiments and the MD simulations represent equivalent hydrodynamic conditions. Thus, despite the difference in length scales, by analysing the system over comparable nematodynamic conditions, we are able to capture the underlying mechanism for cavitation under relevant boundary conditions.

The development of cavitation depends on the local pressure landscape. Figure 3d–f illustrate maps of the local pressure in the *x–y* cross-section of the system. The system at rest exhibits a homogeneous local pressure, as one would expect(not shown here). From the map in Fig. 3d it is clear that this applies to small Ericksen numbers Er≃65 as well. However, as shown in Fig. 3e, for Er≃897 (the onset of cavitation), this situation changes drastically. On the upstream side, the local pressure builds up because of the flow obstruction by the pillar. This is accompanied by a pressure drop on the downstream side, just behind the cylindrical pillar. After passing the constriction the fluid expands freely, and thus resulting in the drop in local pressure. This effect becomes even more pronounced if flow is further increased as shown in Fig. 3f. Notice that within the cavitation area the local pressure equals to the pressure of an ideal gas.

**Figure 4:**
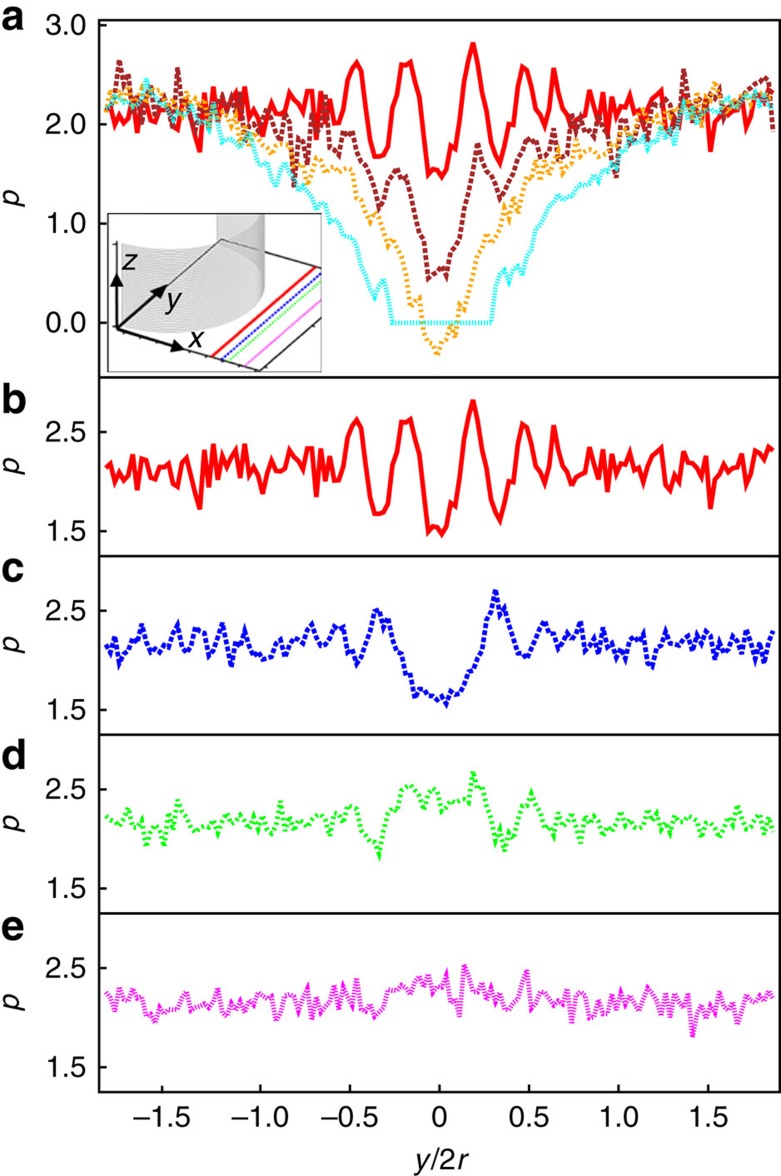
Mapping the local pressure. (**a**) Dependence of the local pressure at distance 0.7 behind the surface of the cylindrical pillar in the direction of flow, plotted across the channel width (*y*-axis) for Er≃65 (red line), Er≃583 (brown dashed line), Er≃786 (orange dashed line) and Er≃897 (cyan dotted line). The inset in (**a**) shows a sketch of different positions behind the cylindrical pillar at which the local pressure was measured. (**b**–**e**) Plots of local pressure at specific distances behind the surface of the cylindrical pillar for Er≃65: (**b**) at a distance of 0.7 behind the pillar, (**c**) at 1.3, (**d**) at 1.7 and (**e**) at 2.7.

Figure 4 presents the corresponding evolution of the local pressure in the vertical plane (*y–z* plane) at specific distances behind the cylindrical pillar. For reasons of symmetry, and to obtain improved statistics, the values of the local pressure have been averaged along the channel depth (*z*-axis), and are presented across the channel width (*y*-axis). To avoid confinement effects, we exclude the regions close to the planar substrates from our calculation. For small flow velocities, the local pressure behind the cylindrical pillar is, on average, constant along the *y*-axis. If flow is increased (see Fig. 4a), the local pressure starts decreasing behind the cylindrical pillar. At the threshold value for cavitation, Er≃786, the local pressure attains negative values. Hence, molecules in this area are pushed out. This occurs just before a cavitation volume nucleates. As soon as a cavitation volume is visible (Er≃897) the local pressure in that area is equal to the ideal gas pressure.

The oscillations visible in Fig. 4a for Er≃65 are due to the layering effects^54^,^55^ of the molecules close to the pillar. This leads to changes in pressure in this region located at the centre of the *y*-axis. If the local pressure is calculated further away from the surface of the cylindrical pillar, layering effects become less prominent, and as shown in Fig. 4b–e, the local pressure minimum (at *y*=0) vanishes. From a molecular standpoint, the local pressure oscillations close to the surface of the cylindrical pillar support the nucleation of a cavitation domain. This is consistent with the fact that hydrodynamic cavitation requires a nucleation site such as a surface.

Figure 3g–i maps the local velocity over the mid-plane cross section (*x*-*y* plane) of the nematofluidic system. At small flow rates, Er≃65, the local velocity is rather low, as indicated by the random orientations of the arrows representing the flow direction (Fig. 3g). At the onset of cavitation, Er≃897, a net flow is clearly present (Fig. 3h). The local velocity in the constriction is considerably higher than that in front of and behind the cylindrical pillar. The relative velocity difference becomes stronger at still higher flows. Figure 3i presents the velocity map at Er≃1,374 in the vicinity of the obstacle and the cavitation domain. Due to lack of sufficient statistics within the gaseous phase, the local convective velocity was set to zero in the cavitation area for calculating the resulting velocity map.

**Figure 5:**
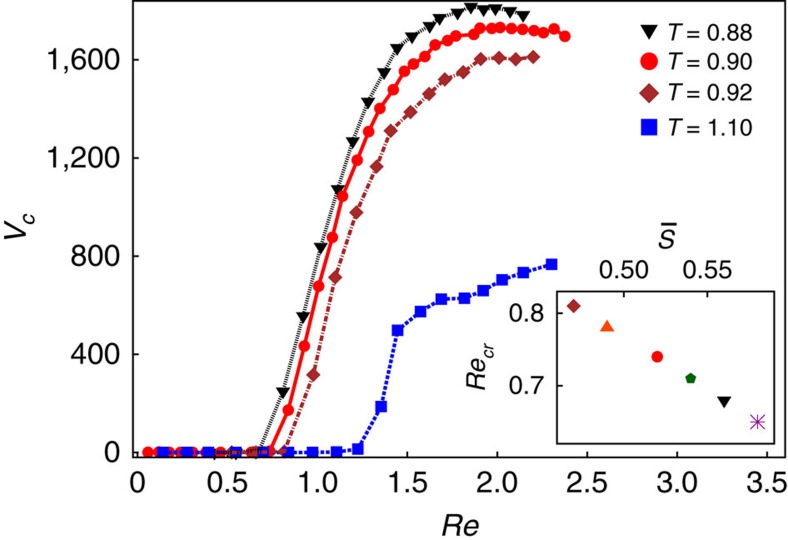
Growth of cavitation volume. Cavitation volume plotted against Reynolds number Re for the nematic LC at *T*=0.88 (black triangle), at *T*=0.90 (red circle) and at *T*=0.92 (brown diamond), and for an isotropic LC (blue square). The inset shows the critical Reynolds number, Re_cr_, plotted against the average nematic order parameter 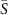, spanning temperatures from *T*=0.92 down to *T*=0.87.

We apply the density maps presented in Fig. 3a–c for estimating the volume, *V*_c_, of the cavitation domain. The total volume was obtained by adding up all the cubic volumes for which the local density *ρ*_l_≤0.15. Figure 5 plots the variation of *V*_c_ with the Reynolds number Re, where Re_cr_ denotes the critical Reynolds number for the cavitation inception. Alongside anisotropic system described so far, we have also performed MD simulations for the same LC model in the isotropic phase (*T*=1.1). The two systems differ in their viscosity *η*: the nematic LC (*η*≃37) is more viscous than the isotropic LC (*η*≃21). Thus, from the definition of Re, it follows that the nematic LC cavitates at lower critical Reynolds number Re_cr_ than the isotropic LC. That fluids with lower viscosity require a larger Re_cr_ to cavitate is consistent with our experiments. Neither the deionized water nor 5CB in isotropic phase elicited cavitation at Reynolds numbers in which the nematic 5CB cavitated. It is important to note here that, in our simulations, the Re_cr_ for the nematic LC is of the same order of magnitude as the local Re in the microfluidic experiments (≃0.1).

For both the nematic and the isotropic phases of liquid crystals, we observe a steep growth of the cavitation domain after its formation, followed by a saturation to a plateau. The height of the saturation plateau is due to the finite size of the simulation box and, therefore, is system size dependent. However, the plateau value of the cavitation domain depends on the nature of the fluid, and can be ascribed to the differences in the liquid matrix structure, for example, different ordering among the three fluids. Figure 5 shows the growth of cavitation domains for the nematic LC at *T*=0.88 and *T*=0.92 that correspond to higher (*η*≃40) and lower (*η*≃34) viscosities, respectively, than what we have considered till now. While the qualitative trend is still retained, quantitative differences emerge, specifically in the growth and saturation of the cavitation domain. The lower the temperature (higher viscosity), the larger was the resulting cavitation volume. In addition to viscosity, the nematic order parameter also varies with *T*. The inset of Fig. 5 shows the dependence of Re_cr_ on the average nematic order parameter 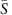 for temperatures ranging from *T*=0.92 down to *T*=0.87. For values of 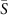 in the nematic phase the critical Reynolds number Re_cr_ decreases linearly with 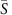, demonstrating that cavitation is enhanced by stronger nematic alignment. This qualitative measurement serves as a first step towards understanding the influence of long range ordering on cavitation at micro scales.

**Figure 6:**
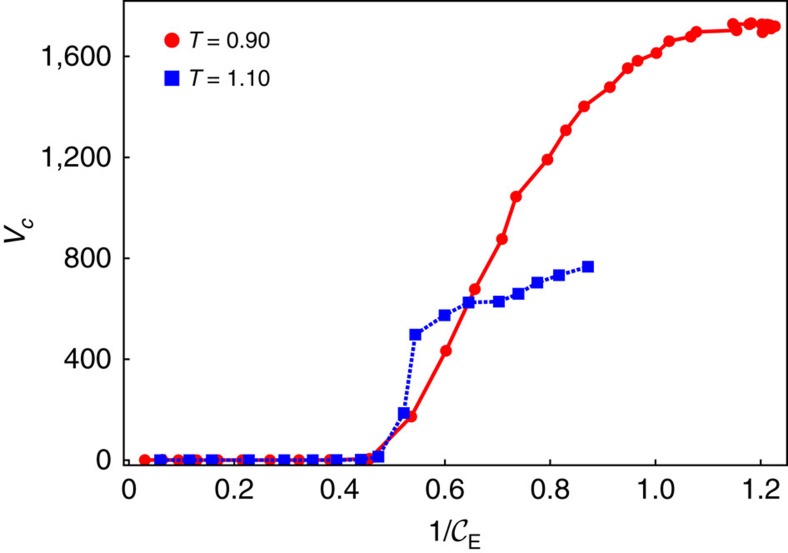
Cavitation volume and Euler number. Dependence of the cavitation volume on the inverse Euler number 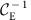 for the nematic LC (red circle) and isotropic LC (blue square).

Figure 6 shows the cavitation volume *V*_c_ in terms of the inverse Euler number 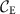, a dimensionless number that is independent of the viscosity of the system. The ratio between the pressure gradient and the kinetic energy per volume is decisive for the development of a cavitating volume, as captured distinctly in Fig. 6. Independent of the model system cavitation occurs at the same Euler number 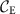. When cavitation first occurs in the experiments we find 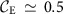 that is within similar order of magnitude as the simulations.

**Figure 7:**
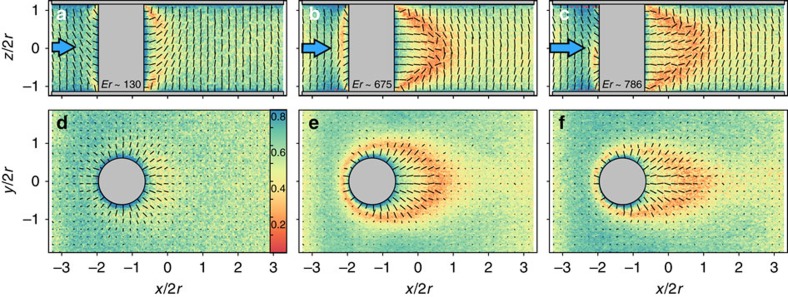
Flow-induced local nematic order. (**a**–**c**) Magnitude of the local nematic order in the *x–z* plane located at *y*=0 (see attached colour bar). The cylindrical grey area represents the cylindrical pillar and the arrow above each plot indicates the direction of flow. Small dashes in the maps represent the local director field such that the length of a dash denotes its three-dimensional orientation: shorter dashes have an orientation closer to the normal to the plane of the map. The Ericksen numbers considered are (**a**) Er≃130, (**b**) Er≃675 and (**c**) Er≃786. (**d**–**f**) Corresponding director fields over the *x–y* plane located at *z*=0.

Figure 7 maps the local nematic order parameter over the range of flow regimes considered here. The flow field reorients the nematic director from homeotropic (on the surfaces) to flow-aligned orientation (in the flowing matrix). Topological defects arise in the director field, close to the top and the bottom walls, where the cylindrical pillar intersects the channel surfaces (Fig. 7a). The singular defect loops are formed due to the homeotropic anchoring, both on the channel surfaces and on the pillar, and are consistent with the defect topology discussed in ref. 49. It is evident from Fig. 7d that over the *x–y* plane, located at the channel half depth (*z*=0), no defect is visible. The director field remains stable for small Er. However, for Er≥675 a single loop around the pillar stabilizes in the *x–y* mid-plane. The loop is deformed and extended towards the downstream direction along with the flow, shown in Fig. 7b,e. Additionally, there is a growing region of flow alignment behind the cylindrical pillar in the downstream direction. Upon increasing Er one can see that the loop becomes stretched further downstream (see Fig. 7c). However, the overall defect topology, especially downstream behind the pillar, is increasingly smeared, possibly due to the appearance of the vapour phase at high Ericksen numbers (Fig. 7f). Changing the surface anchoring from homeotropic to planar did not produce any qualitative change in our results. This agrees well with the experiments, where too we have observed that cavitation in nematic phase was independent of the nature of the surface anchoring.

### Theoretical analysis

We theoretically study the cavitation in an anisotropic matrix and predict the role of nematic order parameter on cavitation inception. As the simplest case, let us consider a spherical environment containing the nematic liquid crystal (see Methods and Supplementary Fig. 1). The fluid is subject to a pressure drop Δ*P*. Under appropriate hydrodynamic conditions, a stationary pressure drop leads to cavitation in the nematic bulk. The first region of interest, the spherical cavitation domain with radius *r*_c_, encloses the vapour phase of the LC. From our foregoing experiments and computer simulations we know that the nematic LC molecules align homeotropically to the surface of the vapour phase. This we consider as the region 2: a spherical shell of thickness *r*_d_−*r*_c_ with perfect homeotropic alignment to region 1. Finally, the rest of the system is the bulk nematic phase that we will treat in the mean-field approximation, namely, with a homogeneous nematic order parameter *S*. Locally, the order parameter may vary as a result of the inception and growth of the cavitation domain. The third region has a radius *r*_L_>*r*_d_, and excludes the sphere of radius *r*_d_.

Although the presence of hydrodynamic flow induces nonequilibrium conditions, the fluid has typically enough time to reach conditions of local equilibrium, and therefore a discussion based on free energies is admissible. We find that the total free energy can be expressed as


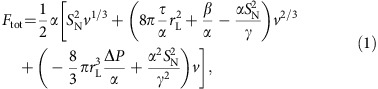


where the cavitation volume 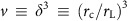, *α*, *β* and *γ* are coefficients with dimensions of energy (see Methods section for details), *τ* the surface tension of the liquid–vapour interface, *S*_N_ is the nematic order parameter in the absence of cavitation and Δ*P* is the absolute value of the pressure drop. Cavitation will occur when the free energy in equation (1) develops a minimum v*>0. This critical point is found from the two conditions *∂F*_tot_/*∂v*=0 and *∂*^2^*F*_tot_/*∂v*^2^=0 that determine the critical cavity volume and critical pressure


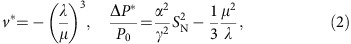


where 
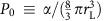
 is a reference pressure, 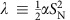, 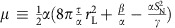. For *λ*>0 and *μ*<0 a finite minimum develops.

The influence of the order parameter on cavitation can be found from studying how v* depends on *S*_N_,


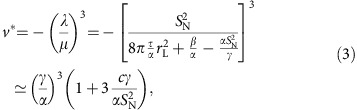


where 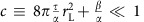. Thus, as *S*_N_ grows, v* decreases and cavitation grows easier. This fact is confirmed by our computer simulations.

The following physical picture emerges from the theory above. Upon increasing flow speeds the pressure in the stagnation point downstream of the obstacle diminishes. The elastic energy associated with the nematic order increases due to the deformations induced by both the flow alignment and the pressure drop. At a certain flow magnitude, it becomes energetically more favourable to produce a liquid–gas interface whereon anchoring conditions compatible with the nematic director alignment can materialize. Consequently, the director field can relax, and the nematic order parameter attains values locally higher and closer to the value in stationary conditions than in the case when no liquid–gas interface would form. We note that the presence of the coupling *α* between order parameter and deformation is crucial to produce nematic cavitation. At vanishing *α*, there is no advantage in producing cavitation. Note also that for vanishing *S*_N_ (that is, not in the nematic phase) cavitation does not occur. Our theoretical arguments are in fact valid only for phases with nematic order.

## Discussion

We report hydrodynamic cavitation in the Stokes flow regime of an anisotropic fluid, due to flow of a nematic LC past a micron-sized pillar within a microfluidic chip. By varying the flow speed within a channel, we studied the growth of a stable, microscopic cavitating domain on the pillar surface that positioned stably at the downstream stagnation point of the micropillar. Cavitating domains were experimentally observed for 200<Er<500, and 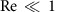, in microchannels possessing both planar and homeotropic boundary conditions. For flows with Er<200, cavitation was not detected, whereas for Er>500, the cavitating domain, after formation, was sheared off due to the high flow speed, and advected downstream. At a given Er, the cavitation domain, over a few hours (5 h for Er=250), attained the saturation volume. By demonstrating that cavitation in complex anisotropic fluids can be triggered at Re≃10^−3^–a hydrodynamic Stokes flow regime, our work complements earlier reports on hydrodynamic cavitation of isotropic fluids at high Reynolds numbers (for example, microchannel flows in deionized water flowing through a micro-orifice by Mishra and Peles^56^,^57^,^58^). The occurrence of cavitation under both planar and homeotropic boundary conditions indicates that the nematofluidic conditions that favour cavitation in 5CB are weakly influenced by the nature of LC anchoring on the microchannel surfaces. This corroborates that the viscous forces play a dominant role relative to the elastic forces, given the high Er at which inception of cavitation is observed in our experiments.

Using MD simulations, we have established a critical Reynolds number criterion, Re_cr_, that sets the threshold Re for cavitation inception. Strikingly, the simulations reveal that the Re_cr_ can be up to 50% lower in anisotropic fluids than in isotropic fluids. While the susceptibility of a liquid to cavitate is clearly linked to its viscosity, importantly still, our finding that Re_cr_ decreases linearly with the global nematic order parameter 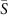 demonstrates that long-range ordering is a key material parameter for cavitation inception in complex anisotropic fluids. Measurements of the growth in cavitation volume revealed that the qualitative behaviour in isotropic LC fluids is similar to the one of anisotropic fluids, for example, the rate of volume growth. However, the size of cavitation domain at large Er numbers is markedly different. This further adds on to the consideration that structural differences of the system, such as long-range order, affect the growth of cavities due to hydrodynamic flow. In addition, our simulations at lower Er numbers (before cavitation sets in) are in very good agreement with earlier microfluidic experiments^49^. Finally, our theoretical calculations support the occurrence of cavitation in nematic LCs, and correctly predict the lowering of the cavitation threshold with increasing nematic order parameter.

Our study is a step towards understanding cavitation in anisotropic fluids that aptly complements investigations on cavitation in isotropic fluids. Beyond its fundamental significance, this study can potentially introduce a novel control parameter for devising applications based on cavitation at low Reynolds number flows and in the development of future biomedical applications.

## Methods

### Materials

We have used 4′-pentyl-4-biphenylcarbo-nitrile, commonly known as 5CB (Synthon Chemicals), as the anisotropic liquid for our experiments. 5CB is a single component LC that is in the nematic phase at room temperature, and was used without any additional purification. In the nematic phase, the 5CB molecules possess a long-range orientational order that is lost at higher temperatures, as 5CB undergoes a transition to the isotropic phase at ∼33 °C (ref. 59). On cooling it down below 18 °C, nematic 5CB undergoes a phase transition to the crystalline phase.

### Microfluidic setup

**Figure 8:**
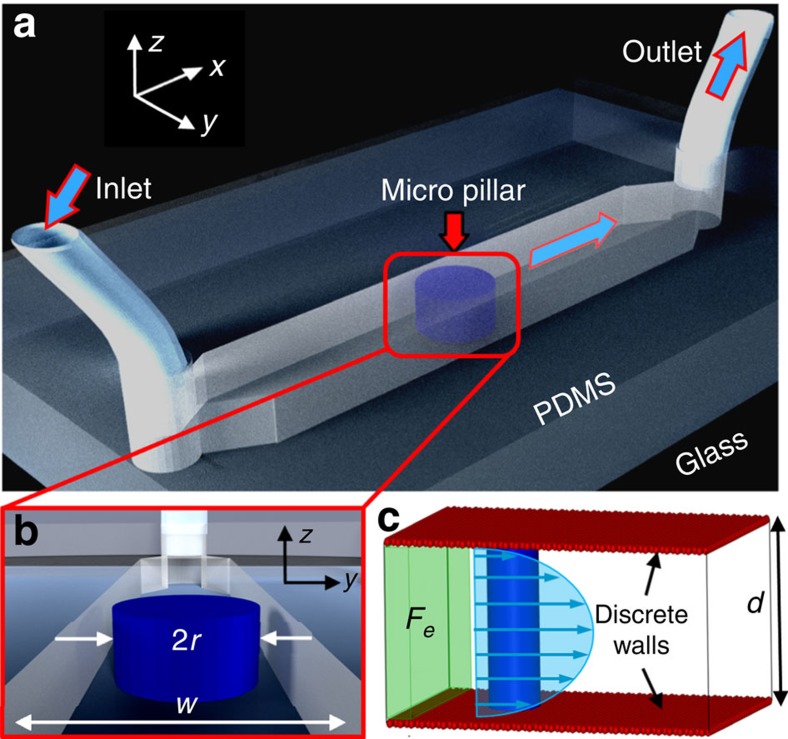
Microfluidic confinement. (**a**) Microchannel with micron-sized cylindrical pillar fabricated using surface bonding of PDMS and glass components. The orthographic projection shows a linear channel of a rectangular cross-section with the integrated micropillar. The *x*, *y* and *z* coordinates denote the flow direction of the anisotropic fluid (a nematic molecule), the transverse direction of the channel (width) and the channel depth, respectively. The blue arrowheads show the flow path. (**b**) Microchannel projection showing the cross-sectional view (*yz* plane) with *r*, *w* and *d* being the pillar radius, channel width and depth, respectively. (**c**) Sketch of the empty simulation channel with the discrete walls.

The microfluidic devices were fabricated using channel reliefs cast on polydimethylsiloxane (PDMS, Sylgard 184, Dow Corning) and surface bonded to glass following exposure to oxygen plasma. The cylindrical pillar was formed as a part of PDMS cast prepared using soft lithography^49^. An orthographic view of the microfluidic chip is shown in Fig. 8a. Experiments were conducted in simple linear channels with a rectangular cross-section and a cylindrical pillar placed at the middle of the channel, spanning the channel depth, *d*, from 10 to 50 μm, and the pillar diameter, 2*r*, between 10 and 80 μm (see Fig. 8b). Here we present results that were obtained (unless otherwise stated) due to the flow of nematic 5CB past micropillars of diameter, 2*r*=80 μm within *w*≈100 μm wide, *d*≈15 μm deep, and sufficiently long (20 mm) channels. In Fig. 8a, *x*, *y* and *z* denote the flow direction, channel width and channel depth, respectively. Cylindrical holes of 750 μm diameter at either ends of the channel served as the housing for the inlet and outlet tubes (Teflon). The tubes (300 μm inner diameter and 760 μm outer diameter) were inserted within the holes punched in the PDMS mould. Smaller diameter of the PDMS housing as compared with the external diameter of the tube ensured good mechanical fitting. Additionally, uncured PDMS was applied at the point of fitting. After curing of the PDMS, any possible leakage at the inlet and outlet ports was ruled out. For filling the channel and subsequent flow experiments, a gear pump (neMESYS, Cetoni) with flow rate precision of nl h^−1^ (±0.02% of the corresponding flow rates) was used to drive a gas-tight microlitre syringe (1001LT, Hamilton Bonaduz), connected to the inlet tube. The outlet tubing was dipped in a sink of 5CB. In agreement with the observed dependence of the swelling ratio on the substance polarity (≃4.8 D for 5CB)^60^, no perceptible swelling of PDMS by 5CB was observed during the course of our experiments. To rule out the presence of pre-existing gas or air pockets entrapped in the surface asperities, and anywhere else in the fluidic circuit, the experiments were conducted after degassing the PDMS-glass microchannels, connected to the LC-filled microfluidic syringe, for 1 h using a vacuum chamber. In addition to visualizing the cylindrical pillar, the entire microfluidic device was periodically scanned to check for other cavitation-like nucleation sites.

### Surface anchoring and flow field characterization

The microfluidic devices employed here comprised two chemically distinct surfaces, namely PDMS and glass. While LC molecules orient perpendicular to the PDMS surface (homeotropic surface anchoring), on the glass surface, the molecules anchor parallel to the glass surface with azimuthal degeneracy (degenerate planar anchoring)^48^. To achieve a homogeneous homeotropic surface anchoring on all the surfaces, the confinement was functionalized using an aqueous solution of octadecyldimethyl(3-trimethoxysilylpropyl)ammonium chloride (DMOAP). The channel was first filled with the DMOAP solution and then rinsed with deionized water (after ≈10 min). The surface treatment was thereafter thermally stabilized by heating the channel at 80 °C for 15 min and at 110 °C for 1 h. This yielded homogeneous homeotropic surface anchoring conditions on all the surfaces. To induce planar boundary conditions, freshly fabricated microchannels were rinsed with a dilute solution of polyvinyl alcohol (average molecular weight=90 k, concentration 0.1% by weight in deionized water). The hydroxyl groups of polyvinyl alcohol bond covalently to silanol groups on the glass surface and plasma-treated PDMS surfaces^59^. The bonding was additionally stabilized by baking the filled channel at 80 °C for 15 min followed by baking at 110 °C for 1 h. On completion of the baking process, the polymer chains, oriented parallel to the confining surfaces, imposed a planar surface anchoring on all four walls of the microchannel.

We have combined white light microscopy in bright-field mode and POM to identify the cavitating domain and the director orientation of the flowing LC molecules, using a Nikon Eclipse LV100 polarizing microscope. Imaging was carried out with a 20 × air objective, NA=0.45. The flow speed was typically extracted from high-speed videos of flowing particles using standard particle tracking techniques^59^. Control experiments, to check for long-term effects of 5CB flow on PDMS surfaces, were also done to specifically identify any surface degradation of PDMS due to 5CB (which can potentially act as a nucleation site for cavitation). We report no surface degradation of PDMS due to flowing 5CB, for at least 24 h of continuous flow.

Light travelling through a singular disclination in liquid crystal can lead to lensing effects that renders the defect optically distinct from the neighbouring ordered region^61^. In high Er nematic flows, disclinations give rise to numerous flowing optical lenses within the flow-aligned nematic matrix. We visualize such disclination lines using video microscopy, close to the micron-scale obstacle. By means of image analysis technique we capture the differential intensity between the frames acquired from the video, and average them over the duration of the video acquisition. Thereby, we obtain a time-averaged differential intensity map of the disclination lines flowing past the obstacle, as shown in Fig. 1c. This information was used as a proxy for the streamlines of the nematic flow, since disclinations—both singular and escaped structures—are stretched due to the viscous drag and, in general, appear as straight lines within the flow^62^.

### Material and nematofluidic parameters

Microfluidic devices, possessing homeotropic and planar surface anchoring, were initially filled with 5CB in the isotropic state, and allowed to equilibrate to nematic phase at room temperature. Thereafter, we progressively increased the volume flow rate *Q* (0.01 μl h^−1^<*Q*<20 μl h^−1^) to detect the first appearance of the microscopic cavitating domain. For a typical channel with dimensions, *w*=100 μm, and *d*=15 μm, the corresponding flow speed, *v*, within the microchannel varied between ≃2 μm s^−1^ and 4 mm s^−1^. Thus, for the nematic LC 5CB having bulk dynamic viscosity, *η*≃50 mPa s^59^, the characteristic Reynolds number Re=*ρvl*/*η* ranged between 10^−6^ and 10^−3^ that, from a hydrodynamic point of view, signifies the Stokes flow regime. Here, *ρ*≃1,025 kg m^−3^ is the material density, and *l*=4*wd*/2(*w*+*d*)≈26 μm is the hydraulic diameter of the rectangular microchannel. The Ericksen number, a dimensionless number that captures the relative importance of the viscous effects over elastic effects in LC flows^44^, is given by Er=*ηvl*/*K*, where *K*=5.5 pN is the single elastic constant of nematic 5CB. It is important to note that the elastic constant does not appreciably change at the constrictions of the microfluidic channel. Elastic constants depend on the nematic order parameter, and there should be no other dependence unless the short-range, molecular structure of the fluid is perturbed. For flow-aligning liquid crystals, the balance of hydrodynamic torque with the elastic one selects a steady-state angle. In this way, the viscous stresses relax to a local equilibrium that does not alter the microphysics of the fluid. We do not expect that the hydrodynamic flow in our experiments, although strong, change the short-range structure of the fluid. From the field map of the nematic order parameter calculated via molecular dynamics simulations and shown in Fig. 7, we can observe that in fact the local order parameter at the constrictions exhibits no particular difference from the values sufficiently away from the micropillar. We can then conclude that the elastic constants will not significantly change in the constrictions.

In our experiments, the corresponding values of Er ranged from ≃0.5 to 850: for Er<1, the elastic effects outweighed the viscous effects, whereas at Er>1, the viscous effects are dominant over their elastic counterpart. As Re and Er are both proportional to *vl*, we immediately find that Re=*Kρ*/*η*^2^ Er. Inserting the typical numbers for the materials properties of LCs, we find a general conversion factor: Re≃10^−4^Er, for *K*≃10^−11^, obtained as a ratio of interaction energy between molecules and the molecular distance, *ρ*≃10^3^ and *η*≃10^−2^ (ref. 44). Therefore, the specific conversion factor depends on the material properties of the particular LC and can vary for different LC compounds. For 5CB, Re≃10^−6^ Er. Nevertheless, the experiments and simulations remain in the laminar flow regime for all Er considered.

### MD simulations

We consider a confined fluid with a cylindrical pillar filled with a fluid composed of *N* molecules (see Fig. 8c). The system is confined by atomically resolved solid substrates that induce friction on the flowing molecules to obtain a steady state during the course of a simulation.

In this work all quantities of interest are given in the usual dimensionless (that is, ‘reduced') units, by taking the molecule's mass *m*, diameter *σ* and potential energy scale *ɛ* as the basic units of mass, length and energy, respectively. All simulations reported here have been carried out for a LC fluid containing *N*=2.4 × 10^4^ molecules in a volume *V*=*s*_*x*_*s*_*y*_*s*_*z*_. We choose a Cartesian reference system oriented as in the experiments. To ensure that the LC fluid is sufficiently deep in the nematic phase we choose a temperature *T*=0.90 and, under isothermal–isobaric conditions, a pressure *P*=1.80. For this thermodynamic state point we obtain a mean number density of 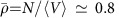 in the bulk.

MD simulations generate molecular configurations by integrating Newton's equations of motion for each particle in the fluid subject to the total potential energy due to other particles, confining substrates and external potentials. The differential equations are solved through a velocity–Verlet algorithm with a time step of *δt*=10^−3^ specialized for LCs^63^. A nonequilibrium, steady-state Poiseuille flow is induced along the *x*-axis by applying a body force 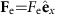 in a thin slice of thickness *δs*_*x*_=2.00 located at *x*=−*s*_*x*_/2+*δs*_*x*_ at one end of the simulation cell and upstream of the cylindrical pillar (see Fig. 8c).

Every simulation is performed according to the following protocol: first, we equilibrate the LC fluid in the isothermal–isobaric (NPT) ensemble without flow; second, a hydrodynamic flow is generated via a pressure gradient and a second equilibration run is performed in the canonical (NVT) ensemble to reach the steady state; finally, we perform the production run in the NVT ensemble, and calculate all physical observables discussed below. For the computation of local quantities we divide our system by means of a grid with cubic cells of side length 0.2.

Because of the external body force, the system needs to be thermostatted permanently to achieve a stationary nonequilibrium state. As explained in detail in previous studies^64^,^65^, the choice of a thermostat capable of efficiently removing viscous heating is of vital importance here because of the presence of the cylindrical pillar. Following our previous work we employ the Galilean-invariant thermostat proposed by Stoyanov and Groot^66^ that, because of its Galilean-invariant nature, conserves momentum locally.

The channel aspect ratio and relative dimension of the microfluidic components were maintained in the simulation domain for MD simulations. Figure 8c shows the sketch of the empty simulation channel with a cylindrical pillar. The channel is confined by discrete walls that are atomically resolved solid substrates that induce friction on the flowing molecules. The green shaded volume marks the region where a body force *F*_e_ is applied to create the Poiseuille flow. We have carried out two sets of simulations: first, where the walls possess homeotropic surface anchoring conditions and, second, where the walls possess planar surface anchoring conditions.

### Static and dynamic observables

We first consider the pressure tensor


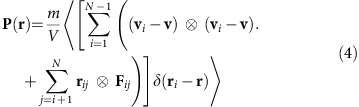


where **v**_*i*_ is the molecule's velocity vector, 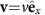 is the averaged local flow velocity, *δ* is the Dirac *δ*-function, ⊗ the tensor product, **r**_*ij*_=**r**_*i*_−**r**_*j*_ the distance vector between the centres of mass of molecules *i* and *j*, respectively, **F**_*ij*_ describes the force acting between those molecules, and 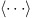 indicates a time average. Notice the convective part of the velocity is subtracted from the kinetic energy contribution in equation (4) and one can therefore apply standard concepts of equilibrium thermodynamics. The scalar pressure is defined by 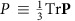. For a gas the contribution of the internal forces in equation (4) becomes negligible. Therefore, the pressure within the cavity is equivalent to the corresponding ideal gas pressure, *P*=*Nk*_B_*T*/*V*.

To analyse the deformation of the director field caused by the presence of the cylindrical pillar and the associated local nematic order we adopt the local alignment tensor defined as^67^


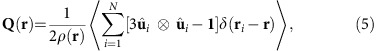


which is a real, symmetric and traceless second-rank tensor. **Q**(**r**) can be represented by a 3 × 3 matrix, where 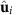 is a versor (unit vector) specifying the orientation of molecule *i* in a space-fixed frame of reference. In equation (5) *ρ*(**r**) is the local (number) density. The local alignment tensor satisfies an eigenvalue equation that we solve analytically. With these solutions we define the local nematic order parameter *S*(**r**) as the largest eigenvalue and the associated eigenvector as the local nematic director 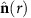.

As in the experiments we characterize the state of the LC fluid under nonequilibrium steady-state conditions with the Reynolds number 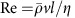, and the Ericksen number Er=*ηvl*/*K*, where we take *l* as the distance between the planar solid substrates. To determine the shear viscosity *η* and the elastic constant *K* of our simulated LC we proceed as in our earlier study^64^.

Finally, to characterize the potential of the LC fluid to cavitate we use the so-called Euler number^68^ 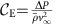 where Δ*P* is the pressure difference between the upstream and downstream end of the simulation box. The Euler number quantifies the ratio of forces due to the local pressure gradient and the kinetic energy per volume.

### Free energy

The total free energy of the system can be written as


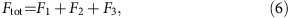


where the indices 1, 2 and 3 refer to the three domains in which the system is divided according to the discussion in the Results section (see Supplementary Fig. 1). The free energy associated to the cavitation domain, region 1, is


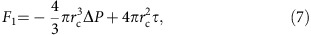


where Δ*P* is the absolute value of the pressure drop and *τ* the surface tension of the liquid–vapour interface. In the following it will be useful to introduce a scaled measure of the cavitation domain *δ*≡*r*_c_/*r*_L_. We can then rewrite *F*_1_ as


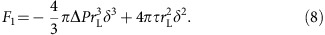


In region 2, the liquid crystal is homeotropically aligned to the surface of the vapour phase. We consider here a perfectly aligned spherical shell of thickness 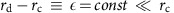. The free energy associated to region 2 stems simply from the Frank free energy (in the one constant *K* approximation)


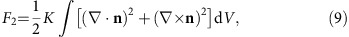


where **n** is the versor representing the director. After integrating, *F*_2_ turns out to be a constant, 8*K*(*r*_d_−*r*_c_), so that it can be ignored in the following.

In region 3 the fluid is modelled as a homogeneous nematic liquid crystal with a constant value of the nematic order parameter. Because of the presence of the cavitation, however, the orientational field of the fluid is deformed. This is due to the fact that the boundary conditions for the nematic alignment are fixed. At the fluid–vapour interface they must be homeotropic, whereas in the bulk of region 3 a homogeneous nematic fluid with a fixed global director is assumed. We consider a mean-field approximation for the treatment of the fluid for *r*>*r*_d_ so that the fluid is characterized simply by the value of its nematic order parameter *S*. This is obviously a simplifying approximation. The stresses induced by cavitation can be relaxed by reorientations of the local director field. We ignore these effects here to keep the theoretical treatment tractable and bring to the front the relevant physics.

With these provisos, we can now build the free energy for region 3. First, there must be a coupling term between the deformation introduced by the cavitation, *δ*, and the global nematic order parameter *S*. At the lowest order in *δ* this must be of the form 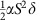. This term reflects the fact that *S* decreases as the relative size of the cavitation increases because the liquid crystal must accommodate two conflicting boundary conditions in an increasingly small volume. The fluid must additionally exert a ‘resistance' to being deformed by the cavitation because of its nematic nature. We model this effect simply with a term ∝*δ*^2^. The last contribution comes from the Landau–de Gennes free energy of a nematic fluid 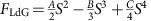, where *A*, *B* and *C*>0.

The ‘deformation' free energy for region 3 can then be written as


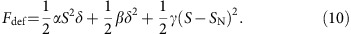


Because the fluid is maintained in the nematic phase, in the last term on the right-hand side of equation (10) we replace *F*_LdG_ with the minimum associated to the nematic phase that is found from the condition *∂F*_LdG_/*∂S*=0, and where the order parameter of the nematic phase 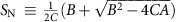 and 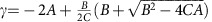. *S* is determined by the deformation *δ*, that is, it is found by minimizing *F*_def_, *∂F*_def_/*∂S*=0. It follows that 
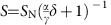
. Equation (10) then becomes


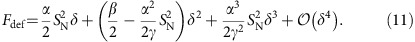


Putting all the pieces together we find equation (1) in the Results section.

### Data availability

The authors declare that the relevant data supporting the findings of this study including raw data and MD simulations custom scripts are available from the corresponding authors on request.

## Additional information

**How to cite this article:** Stieger, T. *et al*. Hydrodynamic cavitation in Stokes flow of anisotropic fluids. *Nat. Commun.* **8,** 15550 doi: 10.1038/ncomms15550 (2017).

**Publisher's note**: Springer Nature remains neutral with regard to jurisdictional claims in published maps and institutional affiliations.

## Supplementary Material

Supplementary Information Supplementary Figure, Supplementary Methods and Supplementary References
