# Supplementary material for: Hydrodynamic cavitation in Stokes flow of anisotropic fluids
Source: Nat Commun. 2017 May 30;8:15550. doi: 10.1038/ncomms15550 (PMC5459993; doi:10.1038/ncomms15550)
Supplement: Supplementary Information — Supplementary Figure, Supplementary Methods and Supplementary References [file ncomms15550-s1.pdf]

Supplementary Methods

Model potential. The total configurational potential energy can be split into three contributions according to

$$\Phi = \Phi_{\rm mm} + \Phi_{\rm ms} + \Phi_{\rm mc}, \tag{1}$$

where the indices refer to interactions among molecules (mm), the interaction between the molecules and the confining substrates (ms), and molecule-cylinder interactions (mc). Assuming pairwise additive interactions in the LC fluid

$$\Phi_{\rm mm} = \sum_{i=1}^{N-1} \sum_{j>i}^{N} \varphi_{\rm mm}\left(\mathbf{r}_{ij}, \hat{\mathbf{u}}_i, \hat{\mathbf{u}}_j\right). \tag{2}$$

The interaction potential $\varphi_{\rm mm}$ is split into an isotropic and an anisotropic contribution according to

$$\varphi_{\rm mm}\left(\mathbf{r}_{ij}, \hat{\mathbf{u}}_i, \hat{\mathbf{u}}_j\right) = \varphi_{\rm iso}\left(r_{ij}\right) + \varphi_{\rm anis}\left(\mathbf{r}_{ij}, \hat{\mathbf{u}}_i, \hat{\mathbf{u}}_j\right), \tag{3}$$

where $r_{ij} = |\mathbf{r}_{ij}|$. We take the isotropic part of the molecule-molecule interaction potential to be given by the Lennard–Jones potential function

$$\varphi_{\rm iso}\left(r_{ij}\right) = 4\varepsilon\left[\left(\frac{\sigma}{r_{ij}}\right)^{12} - \left(\frac{\sigma}{r_{ij}}\right)^{6}\right] \tag{4}$$

where ε is the depth of the attractive well and σ is the van der Waals radius of a spherical reference molecule. Following [1] we write

$$\varphi_{\rm anis}\left(\mathbf{r}_{ij}, \hat{\mathbf{u}}_i, \hat{\mathbf{u}}_j\right) = -4\varepsilon\left(\frac{\sigma}{r_{ij}}\right)^{6} \Psi\left(\hat{\mathbf{r}}_{ij}, \hat{\mathbf{u}}_i, \hat{\mathbf{u}}_j\right), \tag{5}$$

where the anisotropy function is given by

$$\begin{aligned}\Psi\left(\hat{\mathbf{r}}_{\mathbf{ij}}, \hat{\mathbf{u}}_i, \hat{\mathbf{u}}_j\right) &= 5\varepsilon_1 P_2\left(\hat{\mathbf{u}}_i \cdot \hat{\mathbf{u}}_{\mathbf{j}}\right) \\ &+ 5\varepsilon_2\left[P_2(\hat{\mathbf{u}}_i \cdot \hat{\mathbf{r}}_{ij}) + P_2(\hat{\mathbf{u}}_j \cdot \hat{\mathbf{r}}_{ij})\right],\end{aligned} \tag{6}$$

and $P_2\left(x\right) \equiv \frac{1}{2}\left(3x^2 - 1\right)$ is the second Legendre polynomial, $\hat{\mathbf{r}}_{ij} = \mathbf{r}_{ij}/r_{ij}$, and ε_1 and ε_2 are (dimensionless) anisotropy parameters. We take $2\varepsilon_1 = -\varepsilon_2 = 0.08$ throughout this work. The molecules can be described as ellipsoids of revolution with an aspect ratio of 1.26 (see, for example, Fig. 1 of Ref. 2).

The first summand on the right side of Eq. (6) corresponds to the well-known Maier–Saupe term [3]; the other two are corrections describing the orientational dependence of $\varphi_{\rm anis}$ with increased sophistication. This model is capable of forming a nematic phase in a physically realistic fashion [1, 4].

Control over the global nematic director can be achieved by placing the LC fluid between planar, specially prepared solid substrates that serve to anchor molecules at their surfaces in a desired way. Experimentally, this can be achieved either by mechanical means (e.g., rubbing or polishing), deposition of chemical substances on the pristine substrate, or external fields such as, for example, UV [5] or laser light [6] or by exposing the LC to flow [7]. A mathematical "device" that mimics these specially prepared surfaces is the so-called anchoring function g that discriminates energetically between desirable and undesirable orientations of a molecule relative to the substrate plane. In our model, we express $\Phi_{\rm ms}$ in Eq. (1) as

$$\Phi_{\rm ms} = \sum_{i=1}^{N} \sum_{j=1}^{2N'} \varphi_{\rm ms}(r'_{ij}, \hat{\mathbf{u}}_i) \tag{7}$$

assuming that each solid substrate consists of a monolayer of N' atoms. The substrate atoms are located at positions $\mathbf{S} = \{\mathbf{s}_1, \mathbf{s}_2, \ldots, \mathbf{s}_{2N'}\}$, $r'_{ij} = |\mathbf{r}_i - \mathbf{s}_j|$, and

$$\varphi_{\rm ms}(r'_{ij}, \hat{\mathbf{u}}_i) = 4\varepsilon\left[\left(\frac{\sigma}{r'_{ij}}\right)^{12} - \left(\frac{\sigma}{r'_{ij}}\right)^{6} g_z\left(\hat{\mathbf{u}}_i\right)\right]. \tag{8}$$

The atoms in the substrates form two monolayers in registry with each other using the (100) face of a face-centered cubic (fcc) lattice. The fcc (100) structure is characterized by a lattice constant $L/\sigma = \sqrt[3]{4}$ such that the areal density of the solid monolayers corresponds to $\rho_{\rm s} = 2/L^2$. Throughout this work we take the distance $s_{\rm z}$ between both substrate planes to be equal to 20σ.

We choose a homeotropic anchoring parallel to the z-axis

$$g_z\left(\hat{\mathbf{u}}_i\right) \equiv \left[\hat{\mathbf{u}}_i \cdot \hat{\mathbf{e}}_z\right]^2, \tag{9}$$

where $\hat{\mathbf{u}}_z$ is the versor of the z-axis. From Eq. (8) and (9) one realizes that the attractive interaction between a molecule and a substrate atom is switched off if $\hat{\mathbf{u}}_i \perp \hat{\mathbf{u}}_z$ whereas it is fully switched on if $\hat{\mathbf{u}}_i \parallel \hat{\mathbf{u}}_z$. Notice that Eq. 9 is invariant upon replacing $\hat{\mathbf{u}}_i$ by $-\hat{\mathbf{u}}_i$ to comply with the head tail symmetry of the molecules [see Eq. 6].

A cylindrical pillar with a chemically homogeneous surface is located perpendicular to the solid substrates and spans the entire distance between the two substrates. Experimentally, this can be compared to a setup where the cylindrical pillar is part of the cast prepared using soft lithography [8]. We write the molecule-cylinder interaction as

$$\Phi_{\rm mc} = \sum_{i=1}^{N} \varphi_{\rm mc}\left(\mathbf{r}_i, \hat{\mathbf{u}}_i; r_0\right), \tag{10}$$

where $r_0 = 4\sigma$ is the hard-core radius of the cylindrical pillar. To model the molecule-cylinder interaction we adopt the potential function

$$\begin{aligned}\varphi_{\rm mc}\left(\mathbf{r}_i, \hat{\mathbf{u}}_i; r_0\right) = \varepsilon\Bigg[&\left(\frac{\sigma}{r_i - r_0}\right)^{12} - \left(\frac{\sigma}{r_i - r_0}\right)^{6} \\ &\times g_\perp\left(\hat{\mathbf{r}}_i, \hat{\mathbf{u}}_i\right)\Bigg],\end{aligned} \tag{11}$$

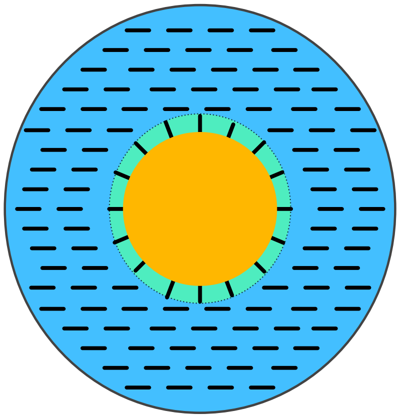

Supplementary Figure 1. **Schematic sketch of the domains considered for the theoretical calculations.** We idealize the microfluidic channel as a spherical environment subject to a pressure drop. The system is divided into three domains: a cavitation bubble (light orange, radius r_c); a thin spherical shell where the nematic director is homeotropically aligned to the surface of the bubble (cyan, thickness $r_d - r_c$); and a 'mean-field' region where the nematic fluid is only slightly perturbed by the presence of cavitation (blue, thickness $r_L - r_d$).

where $r_i = |\mathbf{r}_i|$ and $\hat{\mathbf{r}}_i = \mathbf{r}_i/r_i$. The term $r_i - r_0$ represents the distance of the molecule from the cylinder's surface.

Molecules in the immediate vicinity of the cylindrical pillar also exhibit preferential anchoring because of the specific chemical nature of the cylinder's surface. We consider exclusively homeotropic anchoring, that is, an anchoring locally normal to the cylinder's surface

$$g_{\perp}(\hat{\mathbf{r}}_i, \hat{\mathbf{u}}_i) \equiv [\hat{\mathbf{u}}_i \cdot \hat{\mathbf{r}}_i]^2 \,. \quad (12)$$

Finally, we note that because of the influence of the confining substrates hydrodynamic flow will not produce a complete flow alignment of the molecules with the flow direction. This is due to the small scale of the MD simulations as compared with the mesoscopic scale of the experiments, where flow alignment is easily achieved. We have confirmed this fact with different simulations using different distances between substrates. For larger separations the molecules increasingly align parallel to the flow direction.

Theoretical Analysis. As a first step towards the theoretically study of cavitation in nematic LCs, we need to simplify the geometry of the problem. We thus consider a spherical environment containing a nematic liquid crystal, separated into three different regions. The first region of interest is the spherical cavitation domain, with radius r_c, containing the vapor phase of the LC. Next, we know that the LC molecules align homeotropically to the surface of the vapor-liquid interface. For this, we consider a spherical shell of thickness $r_d - r_c$ with perfect homeotropic alignment. Finally, the rest of the system is a nematic LC which will be treated in the mean field approximation. See Supplementary Fig. 1.

SUPPLEMENTARY REFERENCES

[1] Giura, S. & Schoen, M. Density-functional theory and Monte Carlo simulations of the phase behavior of a simple model liquid crystal. *Phys. Rev. E* **90**, 022507 (2014).

[2] Greschek, M., Melle, M. & Schoen, M. Isotropic–nematic phase transitions in confined mesogenic fluids. the role of substrate anchoring. *Soft Matter* **6**, 1898 (2010).

[3] Maier, W. & Saupe, A. Eine einfache molekular-statistische theorie der nematischen kristallinflüssigen phase. teil i. *Z. Naturforsch. A* **14**, 882 (1959).

[4] Greschek, M. & Schoen, M. Finite-size scaling analysis of isotropic-nematic phase transitions in an anisometric lennard-jones fluid. *Phys. Rev. E* **83**, 011704 (2011).

[5] Reznikov, Y. *et al.* Photoalignment of liquid crystals by liquid crystals. *Phys. Rev. Lett.* **84**, 1930 (2000).

[6] Shan, J., Shi, W., Liu, L. Y., Shen, Y. R. & Xu, L. Optical control of surface anchoring and reorientation of liquid crystals via a plasmon-enhanced local field. *Phys. Rev. Lett.* **109**, 147801 (2012).

[7] Sonin, A. A. *The surface physics of liquid crystals* (Gordon and Breach, Amsterdam, 1995).

[8] Sengupta, A., Pieper, C., Enderlein, J., Bahr, C. & Herminghaus, S. Flow of a nematogen past a cylindrical micro-pillar. *Soft Matter* **9**, 1937 (2013).
